# Adverse childhood experiences and suicidal ideation among nurses in China

**DOI:** 10.3389/fpsyt.2025.1639762

**Published:** 2025-08-18

**Authors:** Ying Ling, Xijie Hou, Jie Zhang

**Affiliations:** ^1^ Department of Nursing, The First Affiliated Hospital of Guangxi Medical University, Nanning, Guangxi, China; ^2^ School of Public Health, Cheeloo College of Medicine, Shandong University, Jinan, Shandong, China; ^3^ Department of Sociology, State University of New York Buffalo State University, Buffalo, NY, United States

**Keywords:** adverse childhood experiences, strain, suicide, nurses, China

## Abstract

**Background and Aims:**

Suicidal ideation and behaviors constitute a serious public health issue both globally and in China, with nurses having a relatively higher suicide rate. While existing research has established that adverse childhood experiences (ACEs) may increase the risk of suicide-related behaviors, the precise mechanisms by which ACEs influence suicidal ideation remain underexplored, particularly among nurses. This research investigates the association between ACEs and suicidal ideation, while also analyzing the mediating effect of psychological strain and the moderating influence of impulsivity.

**Methods:**

Two top public hospitals, one in Southern China and the other Eastern China, were selected to recruit young nurses for study. The sample consisted of 395 individuals with 80.76% being female and aged between 19 and 42 years. A self-reporting questionnaire survey was administered with a computerized program.

**Findings:**

Psychological strain partially mediates the association between ACEs and suicide ideation, with the mediator effect representing 21.70% of the total effect. Impulsivity served as a moderator in the connection between ACEs and suicidal ideation.

**Conclusion:**

This study discloses how ACEs can directly and indirectly influence nurses’ suicidal ideation through psychological strain, and how impulsivity moderates the connection between ACEs and suicidal ideation. The research findings offer significant practical evidence for enhancing nurses’ mental health levels. Future studies could focus on improving nurses’ mental health by addressing factors like mitigating the impact of ACEs, reducing psychological strain, and managing impulsivity.

## Introduction

Suicidal ideation denotes an individual’s contemplation or thoughts regarding suicide, representing a critical phase in the psychological progression toward suicidal actions (1, 2). Suicidal ideation and behaviors constitute a significant public health concern, causing devastating impacts on individuals, families and society (3). According to mortality statistics disclosed by the World Health Organization (WHO), approximately 700,000 individuals globally succumb to suicide annually. This alarming figure underscores the pressing need for prioritizing suicidal ideation prevention as a fundamental component of global mental health strategies (4). The estimated global lifetime rate of suicidal ideation is approximately 9.2%, while the lifetime prevalence of suicide attempts is around 2.7% (5). Suicidal ideation represents the initial phase of suicidal behavior, and individuals who experience such thoughts are at an elevated risk of attempting suicide within the subsequent two years (6). Findings from a national survey in Singapore indicate that the prevalence of non-fatal suicide planning and attempts is 17.7% and 10.6%, respectively. Notably, over 80% of these incidents occur within one year of the onset of suicidal ideation (7). The emergence of suicidal ideation could be a crucial warning sign for potential suicidal behaviors, and the early identification of high-risk populations is of paramount importance in lowering the suicide rate. Hence, exploring the factors contributing to suicidal ideation and developing early warning mechanisms is an important step in improving global public health and promoting mental health.

Adverse childhood experiences (ACEs) refer to the absence of essential stimuli or the existence of threatening stimuli during the normal developmental process of an individual in early childhood, which might have adverse effects on an individual’s lifelong health (8). Individuals who have experienced adverse childhood events show a correlation with increased probability of developing suicidal ideation. The more ACEs an individual undergoes, the higher the likelihood of developing suicidal ideation (9). Research has demonstrated that ACEs, such as parental separation, domestic violence, abuse, and neglect, have been verified to be closely associated with an individual’s mental health issues and may heighten the risk of suicidal ideation by modifying emotional regulation, cognitive patterns, and stress coping mechanisms (10, 11). In recent years, Chinese scholars have increasingly focused on this issue. Research results indicate that childhood adversity experiences have a significant impact on the mental health of children, adolescents, and college students, and are directly associated with their suicidal ideation (12, 13). However, research into the nursing population remains constrained, particularly with respect to the mechanisms by which childhood adversity affects suicidal ideation among nurses, which is still poorly comprehended. Thus, it is essential to carry out thorough research on the connection between ACEs and suicidal ideation in the nursing population to better understand and prevent suicidal behaviors.

Nursing is a profession characterized by significant physical and mental demands, encompassing long and irregular working hours, substantial workloads, elevated stress levels, complex interpersonal relationships, and the risk of burnout. The inherent nature of nursing practice may further heighten the likelihood of nurses experiencing suicidal ideation (14). A cross-sectional study assessing workplace mental health and suicidal ideation among Taiwanese nursing professionals revealed that 18.3% of respondents reported experiencing suicidal ideation within the preceding week (15). A Canadian study revealed that 10.5% of nurses reported experiencing suicidal ideation within the past year, a figure significantly higher than the estimated prevalence in the general population (16). However, the willingness of nurses with suicidal ideation to seek help is significantly lower than that of those without such thoughts, which poses a significant challenge to the effectiveness of current mental health support and suicide prevention interventions (17). Mental health problems frequently stem from an individual’s early life experiences, particularly traumatic ones during childhood (18). Research reveals that nurses exhibit the highest prevalence of depression and anxiety compared to doctors (19, 20). ACEs are correlated with an elevated risk of developing depression, and DNA methylation partially mediates the connection between ACEs and depressive symptoms (21). Anxiety and depression, along with other mental health issues, are closely associated with ACEs. Compared to participants having fewer or no adversities, those experiencing childhood adversities exhibited more severe depression and anxiety (22). Suicide prevention efforts must focus on addressing both individual and systemic factors associated with nurse suicide, such as ACEs or work-related traumatic experiences (23). ACEs might pose latent mental health risks for certain groups of nurses, especially in the high-pressure nursing working environment, where these effects would combine with work stress to heighten the risk of nurses having suicidal ideation. Therefore, it is of vital importance to study the underlying mechanisms of nurses’ suicidal ideation and provide appropriate psychological support to develop effective intervention measures and enhance the mental health of the nurse population, thereby preventing individual suicide incidents.

In the realm of nursing, nurses are not only confronted with a heavy workload but also have to address multiple role conflicts with patients, colleagues, and management, which gives rise to excessive stress in their daily work (24). When stress is not effectively managed, it can evolve into a distinct psychological condition referred to as “Psychological Strain” This phenomenon, also known as “Dissonant Stress,” represents a state of psychological imbalance arising from the interplay of two or more conflicting and opposing factors (25). ACEs could heighten susceptibility to the deleterious effects of stressors encountered in subsequent life, augmenting the negative influence of these stressors and potentially giving rise to suicidal ideation and other adverse health consequences (26). Based on the aforementioned research, psychological strain could serve as a mediator between ACEs and suicidal ideation, functioning as a crucial link between internal and external stressors and eventually resulting in suicidal ideation. Impulsiveness is characterized as a behavioral pattern lacking the ability to adapt to its environment or consequences (27). ACEs may lead to a series of genetic and neurobiological changes, making individuals more susceptible to emotionally dysregulated behavior patterns and having a more intense reaction to stress and negative emotions (28). People with higher levels of impulsivity might have restricted self-control when confronted with extreme emotional distress, making it challenging for them to adopt effective coping tactics and more prone to make impulsive decisions, even manifesting as suicidal ideation or behaviors (29). Therefore, impulsivity might amplify an individual’s response to stress and heighten the risk of suicidal ideation under the influence of ACEs. To sum up, impulsivity could act as a moderating variable, influencing the degree to which ACEs affect suicidal ideation. Although relevant studies have disclosed the relationship between ACEs and suicidal ideation, there is still a lack of sufficient research on the specific mechanisms among the nurse population. However, no research has examined the relationships among ACEs, suicidal ideation, psychological strain, and impulsivity. Hence, this research seeks to examine the connection between ACEs and suicidal ideation among nurses, in the hope of providing significant practical evidence for enhancing nurses’ mental health levels. Based on a literature review, the following hypotheses are put forward for testing:

Hypothesis 1 ACEs have a direct association with the development of suicidal ideation.
Hypothesis 2 ACEs are directly related to psychological strain.
Hypothesis 3 Stronger psychological strain among nurses is correlated with a higher suicidal ideation.
Hypothesis 4 Psychological strain may act as a mediator between ACEs and suicidal ideation.
Hypothesis 5 Impulsivity may function as a moderating variable in the connection between ACEs and suicidal ideation.

**Figure 1:**
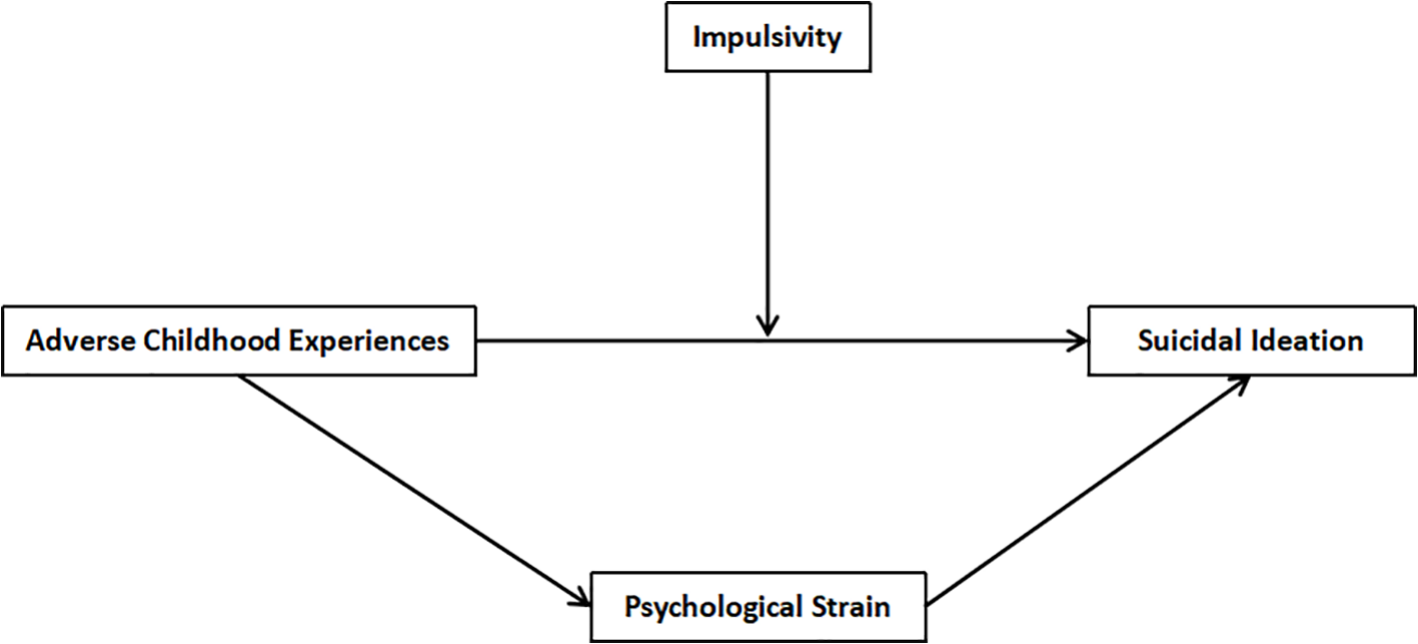
Theoretical hypothesis model.

The theoretical framework illustrating these relationships is depicted in **Figure 1**.

## Methods

### Participants and procedure

Utilizing convenience sampling, nurses from two public hospitals located in the South and East China regions were selected as survey participants between July 2024 and August 2024. The inclusion criteria consisted of the following: working in a local public hospital, possessing a Chinese registered nurse license, and providing informed consent to participate in the study. The exclusion criteria included: nurses who were not employed by the hospital, such as trainee nurses and nursing students. The study utilized an online platform for survey administration. Participants were made aware of the study’s purpose and importance, and they could access the survey by scanning a QR code with their smartphones or computers, followed by entering the corresponding password. Each participant was permitted to complete the survey only once, as determined by their Internet Protocol (IP) address. The study recruited 395 valid respondents and received ethical approval from the Ethics Committee of the First Affiliated Hospital of Guangxi Medical University (2024-E0839). This study complied with the Declaration of Helsinki and obtained informed consent from all participants.

### Sociodemographic information

Utilizing the social population survey questionnaire developed by the research team, we gathered fundamental information regarding the survey participants, encompassing gender, age, education level, average monthly cost of living, exercise frequency, diagnosed with mental illness and seeking help from a psychotherapist.

### Adverse Childhood Experiences

ACEs scale created by Felitti was used to evaluate the ACEs of nurses (30). The psychometric properties of the Chinese adaptation of the ACEs scale have been empirically confirmed through rigorous validation studies, demonstrating satisfactory criterion-related validity and test-retest reliability coefficients (31). The ACEs scale includes three dimensions: abuse, neglect, and dysfunctional family environments. There are a total of 10 items, employing a 2-point scoring system, where “yes” is awarded 1 point and “no” is assigned 0 point, the possible range for the total score is between 0 and 10. A higher score indicates a greater number of ACEs. The reliability coefficient for this measurement is 0.88 (32).

### Suicidal Ideation

The Self-rating Idea of Suicide Scale (SIOSS), developed by Xia et al. (33)was utilized to assess nurses’ suicidal ideation, taking into account the Chinese cultural context. This scale comprised four dimensions, namely despair, optimism, sleep, and masking, encompassing a total of 26 items. Using a 2-point scoring system, where “yes” is assigned 1 point and “no” is assigned 0 point, and reverse scoring is adopted for reverse items, a higher score indicates more intense suicidal ideation. In accordance with the research objectives, this study examines nurses’ suicidal ideation through the application of suicide factors (totaling 4 items). The scale demonstrates robust reliability and validity, as evidenced by a Cronbach’s alpha of 0.79 and a split-half reliability coefficient of 0.814.

### Psychological Strain

The Psychological Strain Scale (PSS), developed by Zhang (25, 34), was employed to assess the psychological strain experienced by nurses. The PSS employs a 5-point Likert response format with 40 items structured across four dimensions: value strain, aspiration strain, deprivation strain, and coping strain. Higher total scores and scores within each dimension indicate an increased level of psychological strain. The scale demonstrates a reliability coefficient of 0.827, with Cronbach’s α values for retest reliability ranging from 0.831 to 0.875 across its dimensions (35).

### Impulsivity

The Dickman Impulsivity Scale was employed to assess the impulsivity of nurses (36). The scale comprises 23 items, split into two sections: 11 items assessing functional impulsive behavior and 12 items evaluating dysfunctional impulsive behavior. A 5-point Likert scale was utilized for scoring, with an internal consistency Cronbach’s alpha of 0.85 for the scale.

### Data analysis

The data analysis was performed using SPSS version 26.0. Firstly, descriptive analysis was conducted to examine the general characteristics of the surveyed nurses, including their exposure to ACEs, suicidal ideation, psychological strain, and impulsivity. For continuous variables, the mean and standard deviation were calculated, while for categorical variables, frequency and percentage distributions were used. Secondly, to ensure the appropriateness of combining samples from the two hospitals, the homogeneity of the nurse populations was assessed in terms of demographic characteristics and key variables. Specifically, independent sample t-tests were employed to compare means for continuous variables, and chi-square tests were used to evaluate distribution differences for categorical variables. Employing the Pearson correlation method to examine the associations among ACEs, suicidal ideation, psychological strain, and impulsivity. Mediation effect analysis was performed using Model 4 of the SPSS macro program PROCESS, while moderated mediation testing utilized Model 5. The standard error of the parameter estimate and the bootstrap confidence interval were obtained by extracting 5,000 bootstrap samples. Significance was determined when the confidence interval did not include the value zero (α=0.05).

## Results

### Characteristics of the Investigation Subject

The final sample comprised respondents from two top public hospitals located in South China and East China, totaling 395 participants. In the hospital in South China, of the nurses, 84.78% were women and 15.22% were men, with an average age of 26.30 ± 3.87 years. Regarding education, 96.20% held a bachelor’s degree or below, and 3.80% had a master’s degree or above. In the hospital in East China, the gender distribution was 77.25% female and 22.75% male, with an average age of 26.06 ± 5.09 years. The educational attainment distribution among the sample population revealed that 96.2% of participants possessed undergraduate qualifications or lower, while 3.8% attained master’s degrees or higher academic credentials, the rest is shown in **Table 1**. There was no significant difference between nurses at both hospitals in terms of gender, age, education level, average monthly cost of living, exercise frequency, diagnosed with mental illness and seeking help from a psychotherapist. Therefore, they will be used as a combined sample for our study.

**Table 1:** Characteristics of participants (N=395).

Variable	Hospitals in South China(N=184)	Hospitals in East China (N=211)	*X* ^2^/*t*	*P*
	f (%) or Mean (s.d.)	f (%) or Mean (s.d.)		
Gender			3.588	0.058
Male	28 (15.22)	48 (22.75)		
Female	156 (84.78)	163 (77.25)		
Age (mean)	26.30 (3.87)	26.06 (5.09)	0.536	0.592
Education level			<0.001	0.995
Bachelor’s degree or below	177 (96.20)	203 (96.20)		
Postgraduate and above	7 (3.80)	8 (3.80)		
Average monthly cost of living (yuan)			1.527	0.466
1300 and below	55 (29.89)	52 (24.64)		
1301-2300	79 (42.93)	101 (47.87)		
Above 2300	50 (27.18)	58 (27.49)		
Exercise frequency			6.736	0.081
Everyday	15 (8.15)	29 (13.74)		
Once a week or more	82 (44.57)	103 (48.81)		
Once a month or more	43 (23.37)	32 (15.17)		
Less than once a month	44 (23.91)	47 (22.28)		
Diagnosed with mental illness			0.771	0.380
Yes	2 (1.09)	6 (2.84)		
No	182 (98.91)	205 (97.16)		
Seeking help from a psychotherapist			0.043	0.835
Yes	5 (2.72)	4 (1.90)		
No	179 (97.28)	207 (98.10)		
ACEs (mean)	0.17 (0.46)	0.53 (1.28)	-3.779	<0.001
Suicidal ideation (mean)	0.11 (0.48)	0.21 (0.57)	-1.973	0.049
Psychological strain (mean)	70.99 (24.06)	79.61 (27.47)	-3.296	0.001
Impulsivity (mean)	61.51 (6.11)	63.66 (6.41)	-3.392	0.001

### Correlation analyses

The relevant analysis outcomes revealed that ACEs had a positive correlation with suicidal ideation and psychological strain; suicidal ideation had a positive association with psychological strain and impulsivity; and psychological strain was positively related to impulsivity. See **Table 2**.

**Table 2:** The correlation analysis of four variables (N=395).

Variables	1	2	3	4
1. ACEs	1			
2.Suicidal Ideation	0.291**	1		
3.Psychological Strain	0.267**	0.298**	1	
4.Impulsivity	0.097	0.142**	0.242**	1

**P<0.01.

### Test of mediating effect

Utilizing the SPSS macro program model 4 developed by Hayes, we examined the mediating role of psychological strain. The findings indicated that ACEs significantly predicted psychological strain and also had a substantial predictive effect on suicidal ideation. Notably, this predictive effect remained significant even when accounting for the mediating variable of psychological strain, as illustrated in **Table 3**. The direct effect size of ACEs on suicidal ideation was measured at 0.303, 95% CI [0.176, 0.431], thereby confirming the significance of this direct relationship. The indirect effect size was 0.084, 95% CI [0.039, 0.149], suggesting a significant mediating function of psychological strain in the connection with ACEs and suicidal ideation, thereby confirming Hypothesis 4. See **Table 4**.

**Table 3:** Testing the mediating model of psychological strain (N=395).

Variables	Suicidal ideation		Psychological strain		Suicidal ideation	
	*β*	*t*	*β*	*t*	*β*	*t*
ACEs	0.388	6.027***	1.751	5.497***	0.303	4.674
Psychological Strain					0.048	4.863
*R^2^*	0.085		0.071		0.137	
*F*	36.326***		30.215***		31.034***	

***P<0.001.

**Table 4:** Analysis of mediating effects of psychological strain (N=395).

	Effect	SE	LLCI	ULCI	Effect size (%)
Direct effect	0.303	0.065	0.176	0.431	78.30
Indirect effect	0.084	0.028	0.039	0.149	21.70

### Test of mediating moderation effect

**Figure 2:**
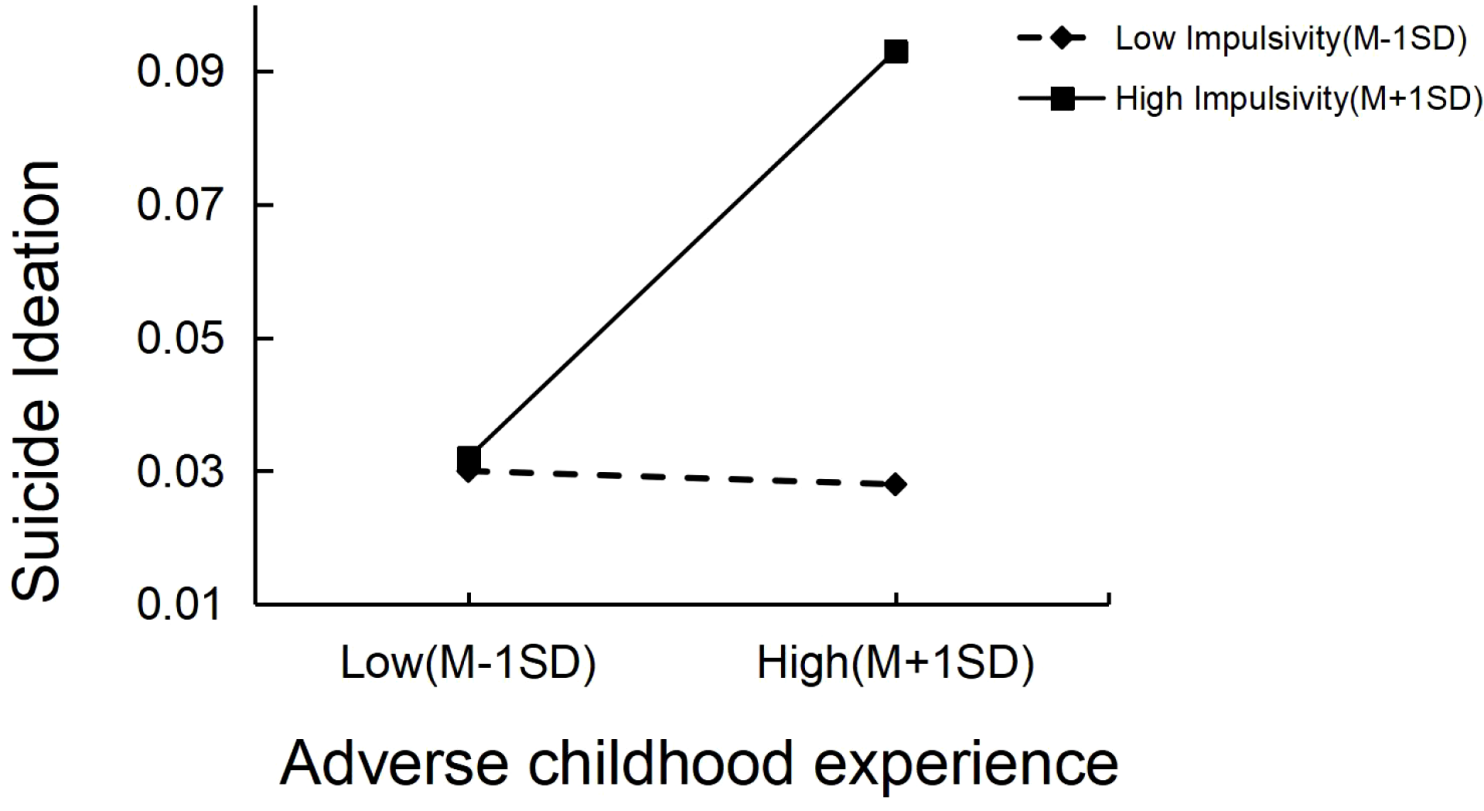
The moderating effect of the impulsivity on the relationship between ACEs and suicidal ideation.

**Figure 3:**
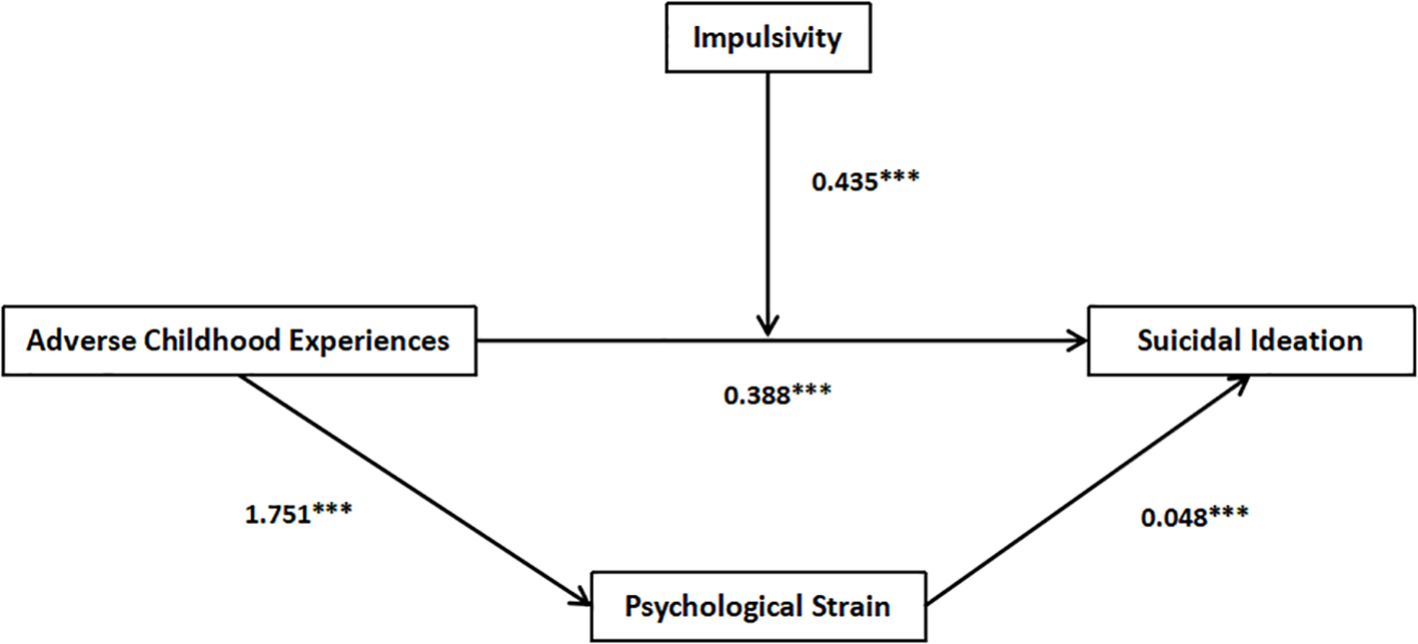
A moderated mediation model. ***P < 0.001.

In this study, the moderating effect of impulsivity on the correlation between ACEs and suicidal ideation was examined utilizing the Model 5 function within the PROCESS macro. The findings indicated that the significant interaction between ACEs and impulsivity predicted suicidal ideation (*β*=0.847, *t*=3.969, *p*<0.001), as presented in **Table 5**. To further clarify the moderating role of impulsivity, simple slope effect plots were generated for impulsivity at standard deviations of +1 and -1, respectively, to analyze the connection between ACEs and suicidal ideation across varying levels of impulsivity, as shown in **Figure 2**. The simple slope test revealed that as ACEs increased, individuals with high impulsivity (*β*=0.45, *t*=6.08, *p*<0.001) displayed a significant upward trend in suicidal ideation, adversity childhood positively predicted suicidal ideation. **Figure 3** shows several important results for adjusting the path coefficients in the mediation model. Hypothesis 5 was supported.

**Table 5:** The moderated mediating effect of impulsivity on ACEs and suicidal ideation (N=395).

Variables	*Estimate*	*SE*	*t*	*P*	*LLCI*	*ULCI*
Constant	-0.045	0.020	-2.281	0.023	-0.084	-0.006
ACEs	0.215	0.067	3.209	0.001	0.083	0.347
Psychological Strain	0.044	0.010	4.462	<0.001	0.025	0.064
Impulsivity	0.033	0.023	1.452	0.147	-0.012	0.078
ACEs × Impulsivity	0.847	0.214	3.969	<0.001	0.428	1.267
*R^2^*	0.174					
*F*	20.571***					

*** *P*<0.001.

## Discussion

Despite a substantial body of research investigating the factors influencing nurses’ suicidal ideation (37–40), there has been a paucity of studies exploring the mediating and moderating roles in this context. This study effectively elucidated the specific intrinsic mechanisms underlying the interaction among nurses’ ACEs, suicidal ideation, psychological strain, and impulsivity. The results indicated that the direct effect of ACEs on suicidal ideation was 78.30%. Furthermore, psychological strain partially mediated the connection between ACEs and suicidal ideation, accounting for an indirect effect that constituted 21.70% of the overall impact. Notably, impulsivity served as a moderator in the connection between ACEs and suicidal ideation. Consequently, the findings of this study are as follows: (a) Nurses’ ACEs were significantly associated with the development of suicidal ideation; (b) Nurses with more ACEs show higher levels of psychological strain; (c) ACEs and psychological strain positively predict suicidal ideation; (d) Impulsivity moderates the connection between ACEs and suicidal ideation.

This study reveals that ACEs exert a significant direct influence on nurses’ suicidal ideation (**Tables 2**, **3**), which is comparable to the findings of previous studies regarding the connection between childhood trauma and suicidal ideation among college students (41). This further substantiates the close link between ACEs and suicidal ideation. ACEs constitute a form of early psychological trauma that profoundly affects an individual’s psychological development and notably enhances the risk of mental health problems including depression in adulthood, thereby influencing an individual’s suicidal ideation (22). As the core workforce of the medical industry, nurses are subjected to high-intensity work pressure. Long-term emotional labor demands, the sense of responsibility for caring for others, and the unstable working environment may give rise to an increased psychological burden. Unresolved traumatic emotions in childhood may intermingle with emotional labor stress in adulthood, thereby augmenting the risk of suicidal ideation. Therefore, medical institutions ought to pay attention to nurses’ mental health issues and enhance mental health training and education. For nurses who have undergone severe adversity childhood, individualized treatment and support should be offered to enhance their overall mental health level and decrease the incidence of suicidal ideation.

This study further discloses that ACEs have an influence on nurses’ suicidal ideation through psychological strain. Psychological strain is an independent risk factor for suicide, and a higher level of psychological stress increases the likelihood of suicide (42). Individuals who have undergone trauma in childhood might be more inclined to develop excessive anxiety, depression, and stress-related disorders when confronted with stress (43). When internal conflicts and sufferings cannot be effectively regulated, individuals may be in a state of intense psychological strain, frequently feeling emotionally out of control or a loss of self-efficacy and being unable to find a solution to the problem. At this point, negative emotional experiences may escalate into suicidal ideation (44). Liu et al.’s research reveals that a significant correlation exists between suicidal ideation and psychological strain among both medical and non-medical staff in Chinese cities, to enhance mental health and guarantee optimal work performance, alleviating psychological strain is of particular significance (45). However, in this study, psychological strain merely offered partial mediating effects, and there might be other unidentified potential mediating effects. Hence, future attention should be directed towards the mental health status of nurses, especially the early intervention for psychological strain. By establishing a comprehensive mental health screening and support system, timely identification and assistance can be provided to nurses to deal with work-related stress and anxiety.

In this research, we discovered that the interaction between ACEs and impulsivity was statistically significant (β = 0.847, p < 0.001), suggesting that impulsivity moderated the connection between ACEs and suicidal ideation. Through the outcomes of the simple slope analysis, we noted that nurses with high levels of impulsivity exhibited significantly stronger suicidal ideation after undergoing more ACEs. This finding is analogous to previous studies; however, previous studies have mainly concentrated on the mediating function of impulsivity in the influence of childhood maltreatment on non-suicidal self-injurious behavior in adolescents (46, 47). In contrast, this study introduced impulsivity as a moderating variable into the model, highlighting its significance in the aspects of nurses’ mental health. One of the causes of impulsive personality traits lies in the impaired decision-making ability. Individuals with such an impairment are more prone to focus on immediate gains while disregarding long-term benefits (48). When confronted with stress, they are inclined to adopt negative coping strategies to escape the current predicament, and negative coping strategies are perilous factors that influence mental health (49). In contrast, highly impulsive individuals might lack rational thinking and coping strategies, and they may demonstrate lower adaptability when dealing with stress. This perspective not only expands our comprehension of impulsive behavior but also offers novel ideas for clinical intervention, particularly in providing mental health support for nursing staff. Hence, in the future, individual impulsive traits should be taken into account in mental health intervention for nursing to achieve more precise intervention strategies. For nurses exhibiting higher levels of impulsivity, it is proposed that regular psychological health evaluations and emotional management training be conducted.

This study elucidates the mediating and moderating roles of adversity childhood experience, suicidal ideation, psychological strain, and impulsivity in their interplay, thereby establishing a theoretical framework for future related research. Nonetheless, this study has its limitations. First, the study has a cross-sectional design, which restricts the capacity to draw causal conclusions between variables. Future studies should adopt longitudinal designs to establish causal mechanisms between these variables through repeated temporal assessments and multivariate confounder control. Secondly, the sample was sourced from public hospitals within a specific geographic region, potentially constraining the generalizability of the findings. Future investigations should replicate this study with a more diverse cohort to enhance external validity. Finally, this study might be prone to self-report bias or potential cultural factors that could have an impact on the results. Future research should lay greater emphasis on the application of a variety of data collection methods, integrating behavioral observation, psychophysiological data, etc. to validate emotional responses and offer a more comprehensive and objective assessment of individuals’ behavior and psychological states. Furthermore, subsequent studies might incorporate additional psychological health variables to evaluate their influence on nurses’ suicidal ideation, thereby offering more practice-relevant recommendations.

## Conclusion

Adversity childhood experiences exert an indirect impact on suicidal ideation via the mediating role of psychological strain. Impulsivity can moderate the connection between ACEs and suicidal ideation. Even with selected control variables and intervention variables, ACEs can still serve as a strong predictor of suicidal ideation. In the modern medical environment, the mental health problems of nurses are increasingly recognized. Paying attention to nurses’ mental health is not only an important measure to safeguard their own health but also a necessary prerequisite for enhancing the quality of medical services. Medical institutions should carry out mental health education and intervention to better ensure the sustainability and effectiveness of medical services.

## Data Availability

The original contributions presented in the study are included in the article/supplementary material. Further inquiries can be directed to the corresponding author.
